# Supplementary material for: Stalled translation on transcripts cleaved by RNase L activates signaling important for innate immunity
Source: bioRxiv. 2025 Jun 11:2025.06.10.658914. Preprint. [Version 1] doi: 10.1101/2025.06.10.658914 (PMC12190479; doi:10.1101/2025.06.10.658914)
Supplement: Supplement 1 [file NIHPP2025.06.10.658914v1-supplement-1.pdf]

bioRxiv preprint doi: https://doi.org/10.1101/2025.06.10.658914; this version posted June 11, 2025. The copyright holder for this preprint (which was not certified by peer review) is the author/funder. This article is a US Government work. It is not subject to copyright under 17 USC 105 and is also made available for use under a CC0 license.

Figure S1

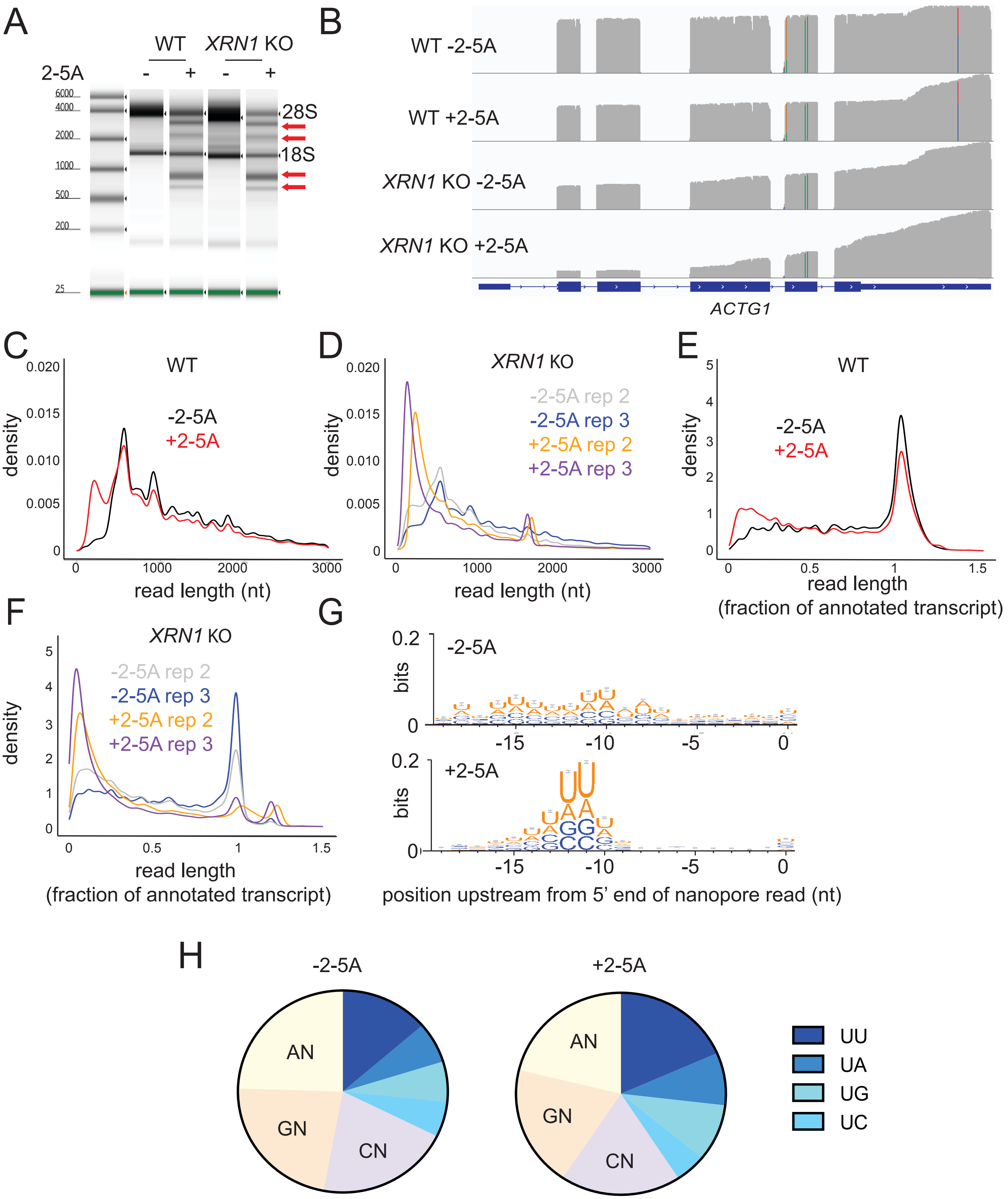

bioRxiv preprint doi: https://doi.org/10.1101/2025.06.10.658914; this version posted June 11, 2025. The copyright holder for this preprint (which was not certified by peer review) is the author/funder. This article is a US Government work. It is not subject to copyright under 17 USC 105 and is also made available for use under a CC0 license.

Figure S1. A rRNA cleavage assays showing RNase L activity in 2-5A treated but not in untreated cells. Red arrows indicate RNase L cleavage products. rRNA cleavage is comparable in WT and *XRN1* KO cells. Image output by Agilent TapeStation software with ladder shown on left. **B** Example gene model (*ACTG1*, oriented 5' to 3') showing a "pile up" view of nanopore raw (not normalized) reads. Image taken from IGV and colored bars indicate mismatches. **C** Length distribution of nanopore direct sequencing reads in 2-5A treated (+2-5A) and untreated (−2-5A) samples in WT cells. **D** Length distribution of nanopore direct sequencing reads in replicate 2-5A treated (+2-5A) and untreated (−2-5A) samples in *XRN1* KO cells. **E** Normalized length distribution of nanopore direct sequencing reads to their respective annotated reference transcript in 2-5A treated (+2-5A) and untreated (−2-5A) samples in WT cells. **F** Normalized length distribution of replicate nanopore direct sequencing reads to their respective annotated reference transcript in 2-5A treated (+2-5A) and untreated (−2-5A) samples in *XRN1* KO cells. **G** Weblogo analysis shows enrichment of U bases in transcriptome positions just upstream of where nanopore sequencing stopped in ±2-5A treated WT cells. **H** Di-nucleotide motif distribution near the 5' end of 3' fragments in ±2-5A treated WT cells (−11-−12 positions as shown in G to account for RNA left in pore after sequencing stops). Total number of fragments in each sample were: 110,068 (-2-5A), 619,964 (+2-5A). In both G and H, 3' fragments were defined as reads that were shorter than a third of their respective annotated transcript.

bioRxiv preprint doi: https://doi.org/10.1101/2025.06.10.658914; this version posted June 11, 2025. The copyright holder for this preprint (which was not certified by peer review) is the author/funder. This article is a US Government work. It is not subject to copyright under 17 USC 105 and is also made available for use under a CC0 license.

Figure S2

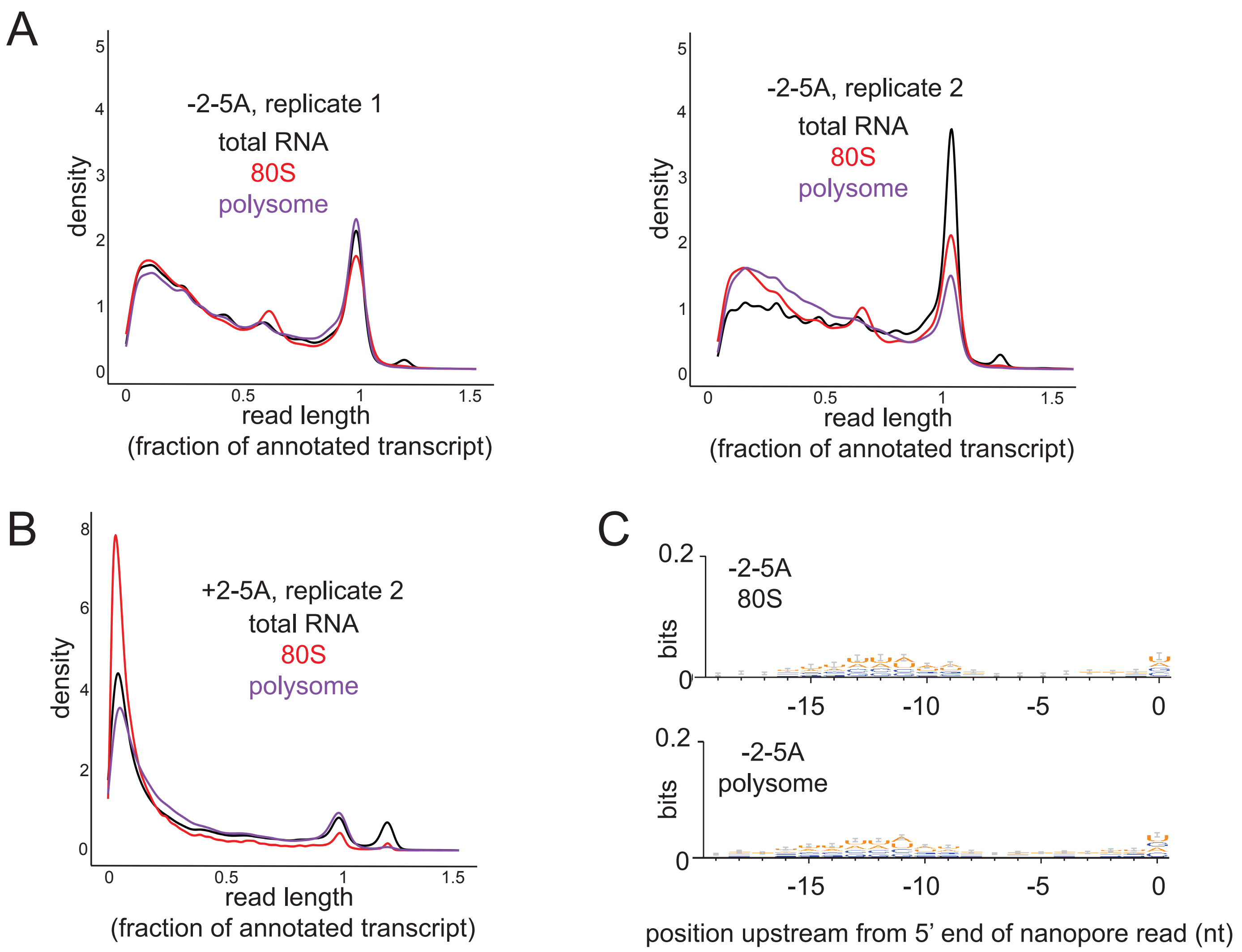

bioRxiv preprint doi: https://doi.org/10.1101/2025.06.10.658914; this version posted June 11, 2025. The copyright holder for this preprint (which was not certified by peer review) is the author/funder. This article is a US Government work. It is not subject to copyright under 17 USC 105 and is also made available for use under a CC0 license.

Figure S2. A Normalized length distribution of nanopore direct sequencing reads to the annotated reference transcript in total RNA, monosomes and polysomes in untreated *XRN1* KO cells (replicate 1 and 2). **B** Replicate experiments for Figure 2C. **C** Weblogo analysis shows enrichment of U bases in transcriptome positions just upstream of where nanopore sequencing stopped in the 80S and polysome fractions of sucrose gradient sedimentation experiments in untreated *XRN1* KO cells.

bioRxiv preprint doi: https://doi.org/10.1101/2025.06.10.658914; this version posted June 11, 2025. The copyright holder for this preprint (which was not certified by peer review) is the author/funder. This article is a US Government work. It is not subject to copyright under 17 USC 105 and is also made available for use under a CC0 license.

Figure S3

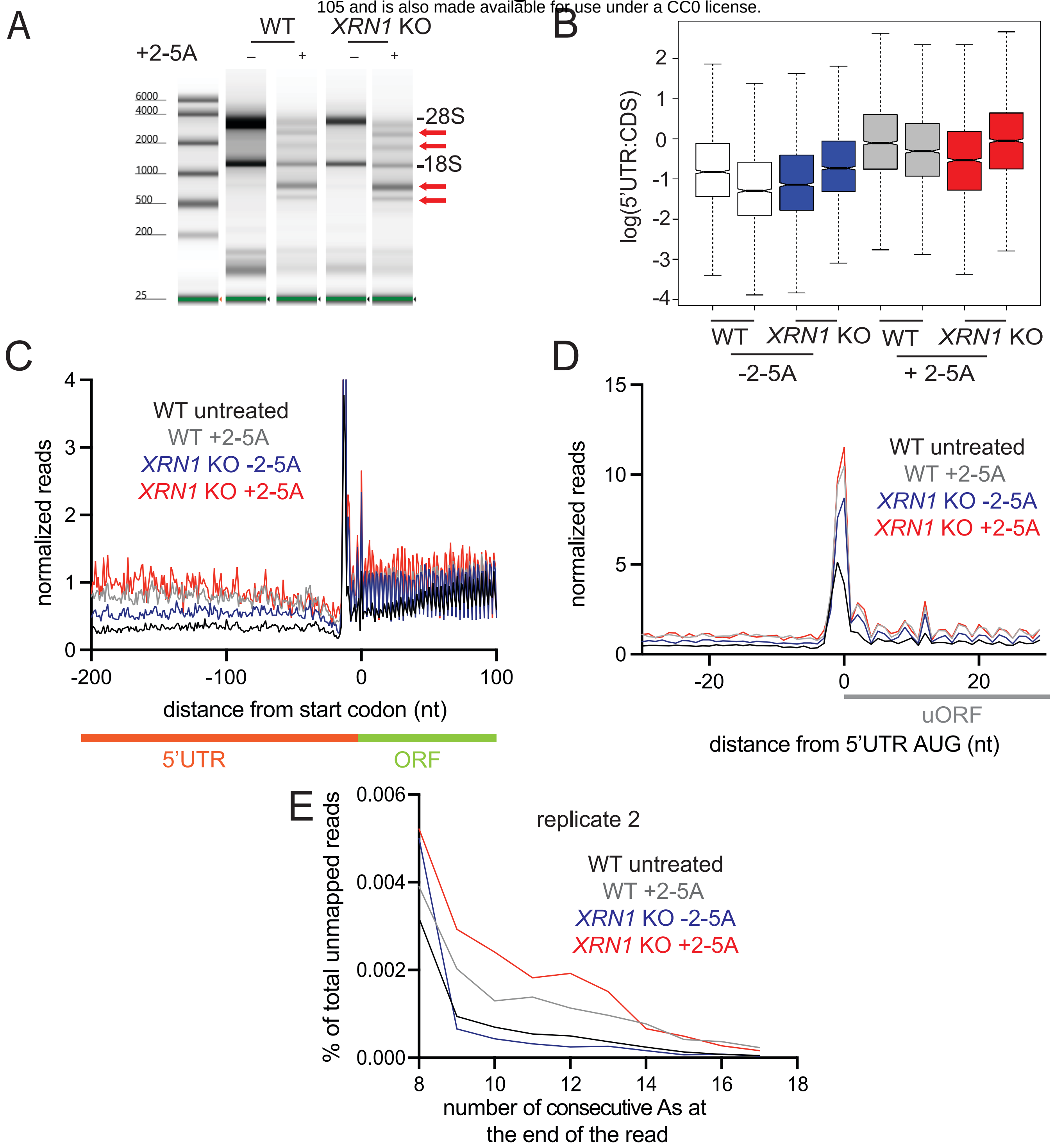

bioRxiv preprint doi: https://doi.org/10.1101/2025.06.10.658914; this version posted June 11, 2025. The copyright holder for this preprint (which was not certified by peer review) is the author/funder. This article is a US Government work. It is not subject to copyright under 17 USC 105 and is also made available for use under a CC0 license.

Figure S3. A rRNA cleavage assays in lysates used in ribosome profiling showing RNase L activity in 2-5A treated but not in untreated cells (replicate 1). Red arrows indicate RNase L cleavage products. rRNA cleavage is comparable in WT and *XRN1* KO cells. Image output by Agilent TapeStation software with ladder shown on left. **B** 5’UTR:CDS ratios do not increase in treated (+2-5A) and control (−2-5A) *XRN1* KO as compared to WT cells. **C** Normalized average ribosome footprint occupancy (metagene plot) around the start codon of main ORFs (CDSs) reveals increased relative ribosome footprint levels in 5’ UTRs when RNase L is active vs −2-5A control. XRN1 KO cells don’t exhibit increase in 5’UTR footprints as compared to WT (red). Ribosome footprints are plotted by 5’ assignment without any shift. **D** Normalized average ribosome footprint occupancy around the start codon of upstream ORFs in the 5’UTR reveal increased uORF translation during RNase L activation. This is not further increased in *XRN1* KO cells as compared to WT cells. Ribosome footprints are plotted by 5’ assignment shifted by 12 nt (~P site). **E** Percentage of ribosome profiling footprints in samples prior to mapping that contained consecutive poly(A) sequences (8-17 nt) at their 3’ end in WT and *XRN1* KO cells (replicate 2). These represent ribosomes that run into the poly(A) tail. The effect increases when XRN1 is absent.

bioRxiv preprint doi: https://doi.org/10.1101/2025.06.10.658914; this version posted June 11, 2025. The copyright holder for this preprint (which was not certified by peer review) is the author/funder. This article is a US Government work. It is not subject to copyright under 17 USC 105 and is also made available for use under a CC0 license.

Figure S4

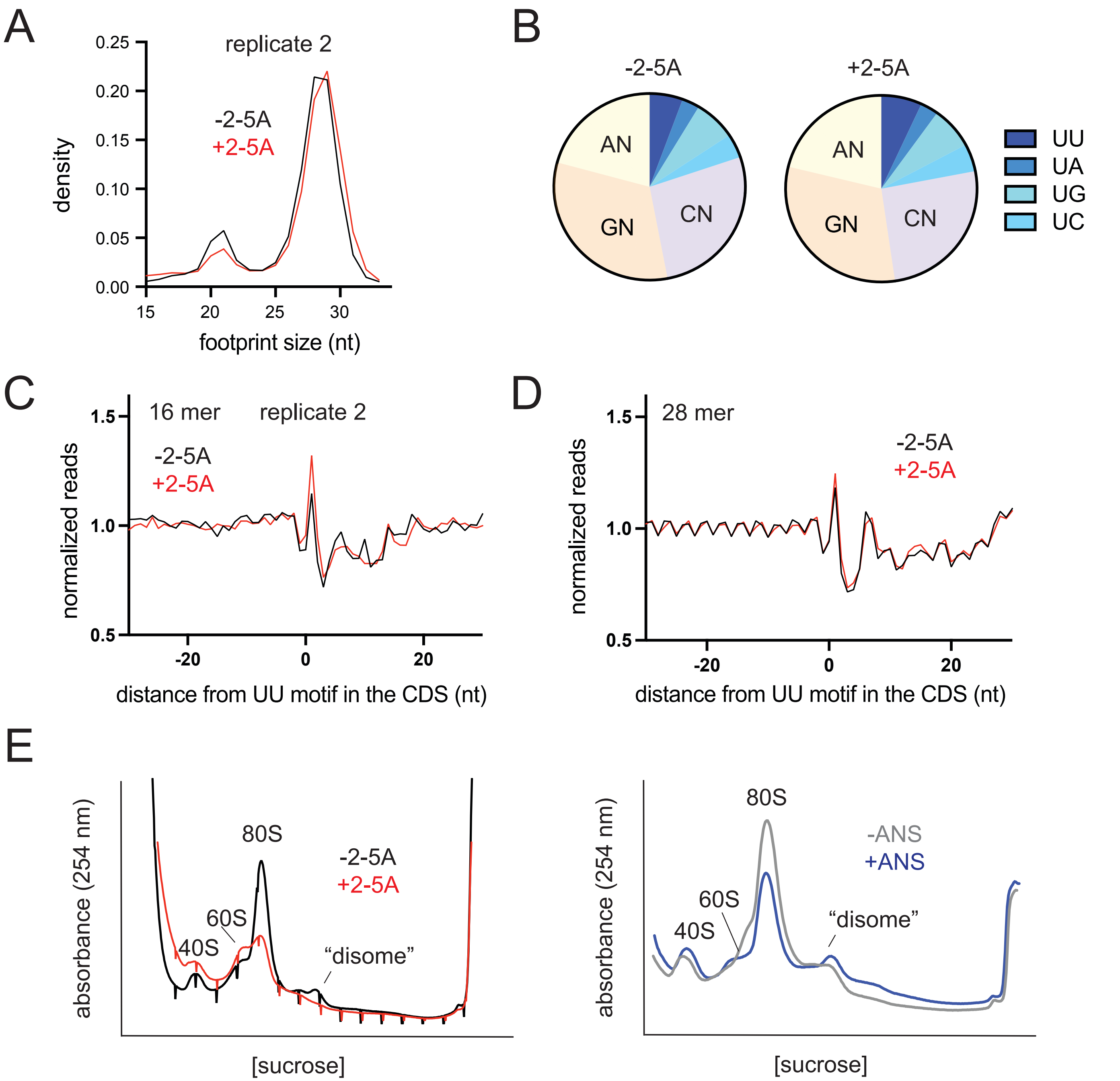

bioRxiv preprint doi: https://doi.org/10.1101/2025.06.10.658914; this version posted June 11, 2025. The copyright holder for this preprint (which was not certified by peer review) is the author/funder. This article is a US Government work. It is not subject to copyright under 17 USC 105 and is also made available for use under a CC0 license.

Figure S4. A Normalized length distribution of transcriptome mapped ribosome profiling reads (15-34 nt) for replicate experiments (replicate 2). **B** 3' end di-nucleotide motif distribution in 16-mer reads. Proportion of short footprints (15-18 nt) containing UU motif at their 3' end is modestly increased in 2-5A treated cells (replicate 2). **D** and **C** Position average plot of 3' ends of 16-mers and 28-mers at RNase L cleavage sites (UU) for replicate experiments. Ribosome footprints are plotted by 3' assignment. **E** RNase A treatment of the lysates combined with 10-35% sucrose gradient ultracentrifugation serves as an assay for ribosome collisions with ANS serving as a positive control. The level of RNA was monitored by absorbance at 254 nm during fractionation.

bioRxiv preprint doi: https://doi.org/10.1101/2025.06.10.658914; this version posted June 11, 2025. The copyright holder for this preprint (which was not certified by peer review) is the author/funder. This article is a US Government work. It is not subject to copyright under 17 USC 105 and is also made available for use under a CC0 license.

Figure S5

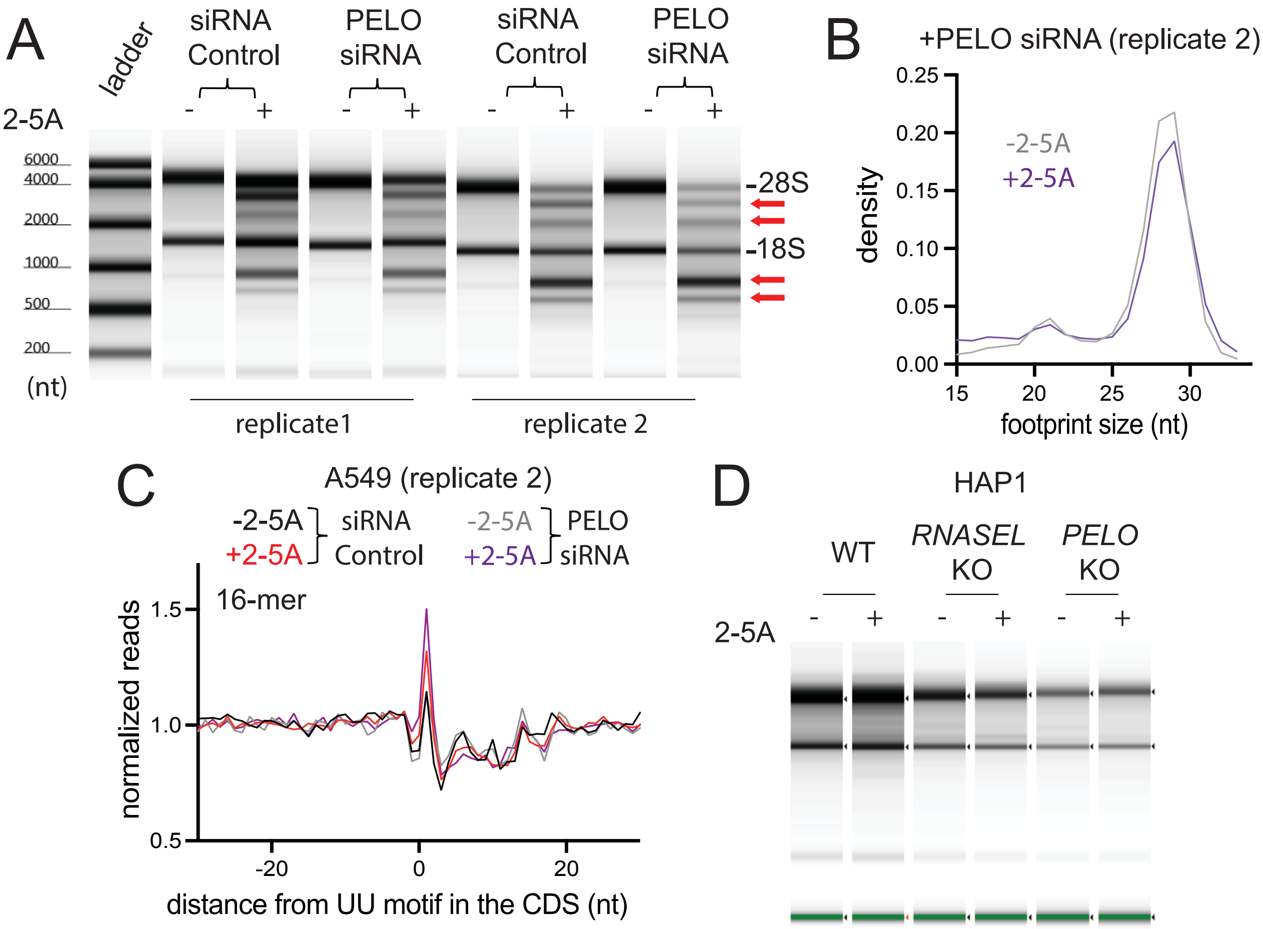

bioRxiv preprint doi: https://doi.org/10.1101/2025.06.10.658914; this version posted June 11, 2025. The copyright holder for this preprint (which was not certified by peer review) is the author/funder. This article is a US Government work. It is not subject to copyright under 17 USC 105 and is also made available for use under a CC0 license.

Figure S5. A rRNA cleavage assays from lysates used in the 15-34 nt ribosome profiling experiments where PELO was knocked down show RNase L activity in 2-5A treated but not in untreated cells. Red arrows indicate RNase L cleavage products. rRNA cleavage is comparable across samples. **B** Normalized length distribution of transcriptome mapped ribosome profiling reads (15-34 nt) in PELO siRNA treated cells (replicate 2). **C** Position average plot of 3' ends of 16-mers near RNase L cleavage motif (UU) in PELO KD A549 cells (replicate 2). **D** rRNA cleavage assays in HAP1 WT and PELO KO cells shows little RNase L activity.
